# A Prolonged History of Denture Impaction for 12 Weeks In Situ

**DOI:** 10.7759/cureus.26002

**Published:** 2022-06-16

**Authors:** Kaso Ari, Syed Zohaib Maroof Hussain, Abdul Wadood Mohammad, Ramez Nassif

**Affiliations:** 1 Education, Norfolk and Norwich University Hospital, Norwich, GBR; 2 Department of Otolaryngology, Head and Neck Surgery, Norfolk and Norwich University Hospital, Norwich, GBR

**Keywords:** ct neck, laryngoscope, oesophago-gastro-duodenoscopy, foreign bodies airway, denture

## Abstract

Foreign body ingestion has serious consequences if left untreated. Impacted dentures for a prolonged period can lead to life-threatening complications. Therefore, prompt diagnosis and immediate intervention are lifesaving. Our patient presented to his local accident and emergency department after having swallowed his dentures during a meal. Initial investigations and workup detected no abnormalities and he was discharged back to the community. Twelve weeks following ingestion, he had developed dysphagia and weight loss which prompted an urgent referral for oesophago-gastro-duodenoscopy (OGD). This identified the dentures impacted within the upper oesophagus and initial attempts at removal were unsuccessful, therefore he required hospital admission for alternative feeding in the interim. A joint procedure with the Ear, Nose and Throat and upper gastrointestinal surgeons was carried out to successfully remove the dentures endoscopically. The patient made an immediate recovery, resuming his normal oral diet with appropriate follow up after discharge. It is suspected our patient had an impacted denture for a period of 12 weeks without sustaining any life-threatening complications, which makes this case rather unique. This case highlights the importance of thorough and careful clinical history taking and examination.

## Introduction

Foreign body (FB) ingestion with upper cervical oeosphageal impaction is common in elderly edentulous patients and those with neurological or psychiatric illness [1]. Dentures as an ingested FB poses diagnostics difficulties due to the radiolucent material it is composed from when using plain radiological imaging to identify it. Patients with impacted dentures in the upper oesophagus commonly present with dysphagia, odynophagia or non-specific pain in the neck or chest [2]. Consequently, this can lead to a delay in diagnosis and management which can lead to complications such as oeosphageal perforation, diverticulum or fistula formation. In addition, the rate of complication of impacted dentures within 24 hours of ingestion is 3.2%, with a dramatic increase to 23.5% after 48 hours [3], whereby those aged >60 years and duration of oeosphageal impaction >24 hours have been shown to be independent risk factors for FB-related complications [4].

## Case presentation

A 64-year-old gentleman presented to Accident and Emergency (A&E) after accidentally swallowing his dentures during a meal. In this visit, clinical assessment and plain imaging did not detect any abnormalities and the patient was subsequently discharged home. Over time, he had developed progressive dysphagia, odynophagia and weight loss with multiple presentations to General Practice (GP) and re-admission to A&E. After 12 weeks from first presentation he was referred to an outpatient clinic under the general surgical team, whereby an urgent oesophago-gastro-duodenoscopy (OGD) was carried out and had first detected the dentures located in the upper oesophagus at the level cricopharyngeus. Due to the shape of the dentures, the edges were impacted in the mucosa of the oesophagus and attempts to remove it during initial OGD were unsuccessful. Consequently, he required emergency admission under the ear, nose and throat (ENT) team for further investigations and management. The patient was otherwise clinically stable, he denied any chest pains or neck pains likely attributable to FB-related complications especially given the prolonged history of oesophageal impaction. Examination of the throat and neck demonstrated no obvious mass or tenderness on palpation. 

**Figure 1:**
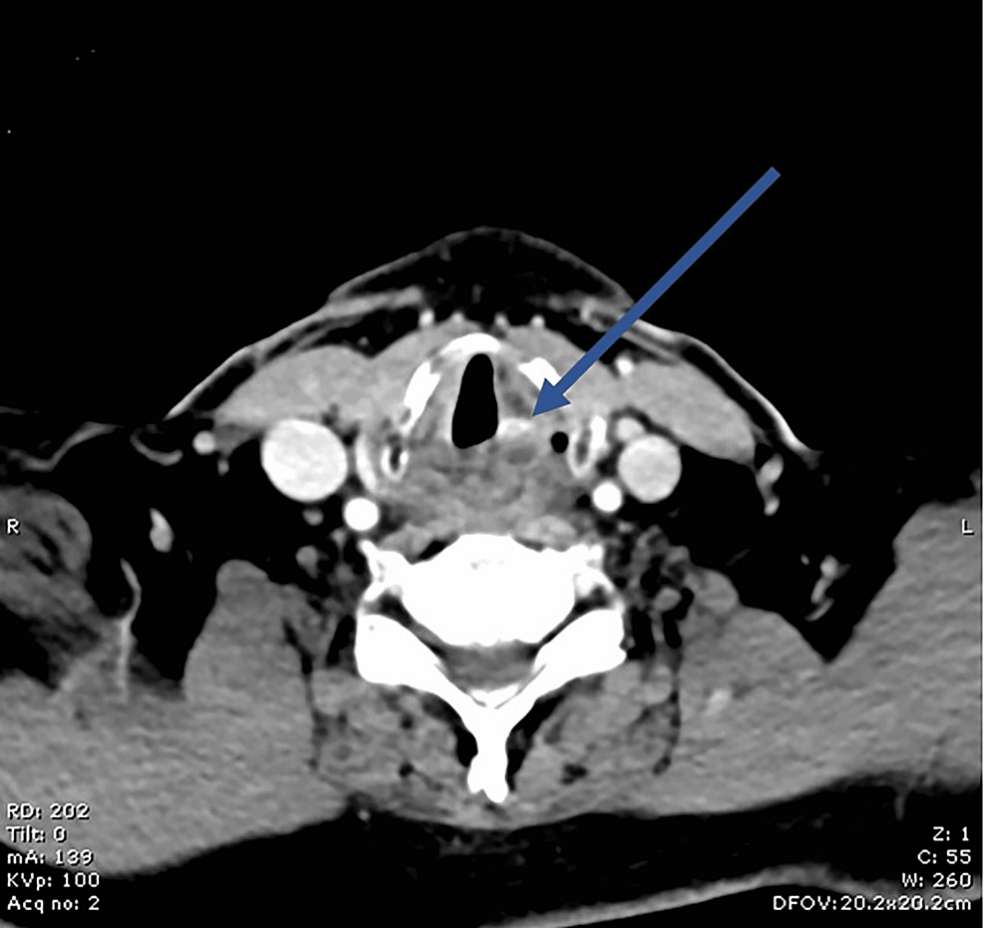
CT neck with contrast image at C6 level showing high-density foci within the post-cricoid region (arrow)

On admission, laboratory blood tests demonstrated normal levels of inflammatory markers, including C-reactive protein (CRP), and normal renal function with an estimated glomerular filtration rate (eGFR) of >90. Chest X-ray did not show any signs of a pneumomediastinum or aspiration pneumonia. A computerized tomography (CT) scan reported a high-density foci within the post-cricoid region with inflammatory changes of the constrictor muscles and inferior portion of the cervical oesophagus (Figure 1).

A water-soluble contrast swallow study showed a 2 cm filling defect in the proximal oesophagus on the right side with no oeosphageal perforation. 

The patient was made nil-by-mouth on admission, commenced on intravenous fluid therapy and prophylactic antibiotics (intravenous co-amoxiclav) for potential risk of oesophageal perforation and mediastinitis. Initially, he underwent an emergency rigid oesophagoscopy to remove the dentures under general anaesthesia. This had further proved ineffective, due to the position of the FB. The two artificial teeth were visualised protruding from the cricopharyngeal inlet, however the wing-shaped plate was tightly impacted just below the cricophraynx.

**Figure 2:**
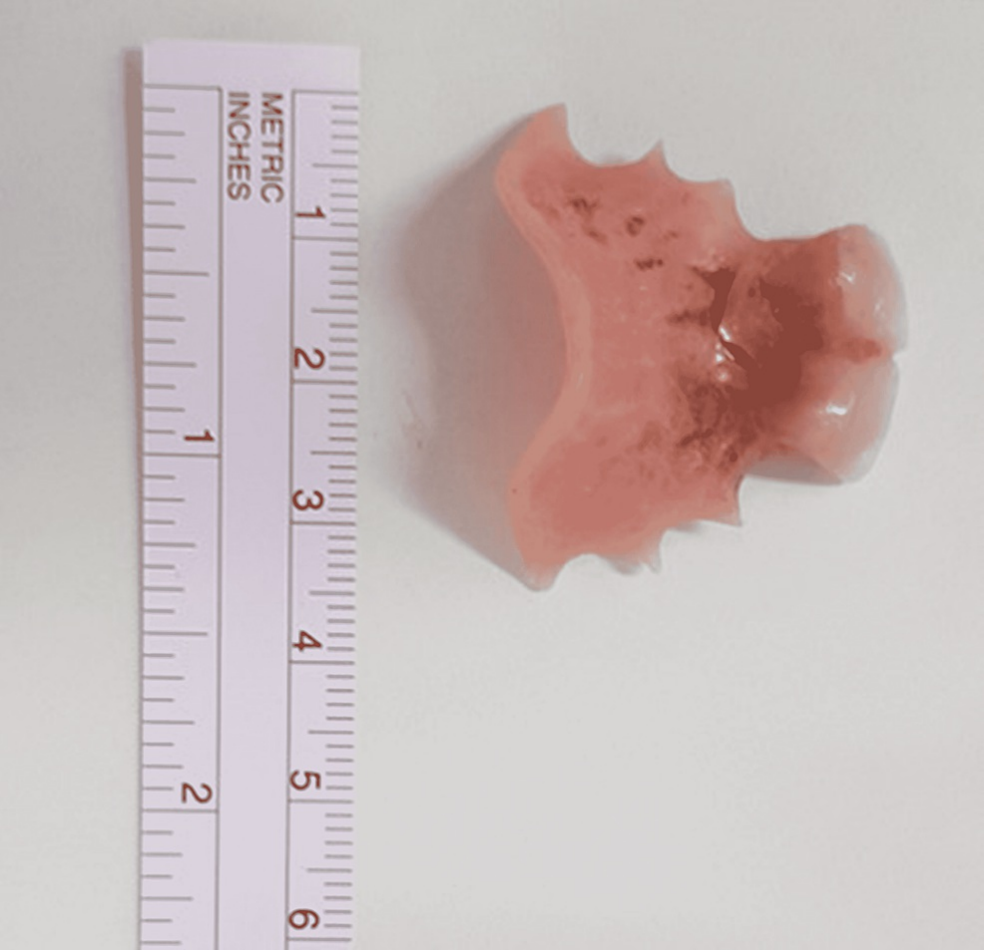
Denture size and shape

This was not completely visualised, it could not be dislodged and further manipulation was causing bleeding. Hence the procedure was aborted, and the patient extubated. It was determined the dentures had caused multiple abrasions to the gastrointestinal (GI) tract which inadvertently led to mucosal overgrowth on the dentures, further making removal difficult without risk of perforation. Post-procedure contrast swallow was done to rule out perforation. Further discussion was made with the upper-GI surgeons and a plan was made to remove the denture by external oesophagotomy if rigid oesophagoscopy failed on second attempt. Under general anaesthesia a flexible oesophagoscopy and removal was attempted. However, the forceps could not grip the denture properly and the attempt failed. Then, rigid oesophagoscopy along with rigid Hopkins rod endoscope was used to visualise the denture. Under maximal muscle relaxation, we were able to mobilise one end of the denture plate and pull it through the cricopharynx, essentially rotating the denture vertically and removing it (Figure 2). 

Nasogastric tube was inserted. Post procedure there were no clinical signs of perforation or mediastinitis. Contrast swallow on day two confirmed that there was no perforation. The patient was started on clear fluids and gradually increased consistency. He was discharged on day three.

Six weeks post discharge, the patient is eating and drinking well without swallowing difficulties or gastrointestinal-related issues. He is due for a clinic appointment under the upper-GI team.

## Discussion

FB ingestion in the upper digestive tract is a common pathologic condition in otolaryngology. It is particularly common in children [5], prisoners, and psychiatric patients [1]. Most ingested foreign bodies pass through the digestive tract uneventfully. However, migration in the aero-/digestive tract can lead to significant morbidity and, in some cases, mortality [6]. Dentures as an FB are not an uncommon presentation and account for 11.5% of impacted FBs in the trachea or oesophagus [7]. Denture ingestion can occur due to many reasons including trauma, intoxication, loss of consciousness or during sleep [8]. Other causes of ingestion include ill-fitting dentures, damaged dentures and seizure patients [9]. 

Identifying denture ingestion can be a challenging task, therefore thorough and careful history taking, and clinical examination are essential. Elderly patients pose a particularly difficult challenge as they are often unaware of the denture ingestion, and the radiolucent nature of denture material means plain film imaging is of no benefit [2]. In addition, if patients remain asymptomatic, as is often the case, diagnosis is delayed. Dysphagia and tracheal tenderness are the common presenting complaints in case of denture ingestion [2]; our patient presented with a history of progressive dysphagia as well.

He was aware of the denture ingestion and presented to A&E with query denture ingestion but given X-ray imaging was normal and the patient was asymptomatic, he was discharged from the department without any further follow-up. According to the medical records and normal plain radiological findings when presenting to the ED during the initial few months, the symptoms did not instigate the need for further invasive investigations or referral to ENT from the emergency physicians. 

Later, he developed progressive dysphagia and re-presented multiple times for the same complaint. He was subsequently referred for an OGD procedure which showed denture impaction in the oesophagus at the level of the cricopharynx. Plain film imaging is the first line of investigation in case of FB ingestion and is useful in identifying steak bones and showing signs of perforation such as free mediastinal and peritoneal air [8,10]. However, negative plain film radiographs do not exclude the presence of FB because fish bones, chicken bones, plastic, wood, glass and thin metal objects are not readily visible [10]. In addition, radiolucency of dentures makes this more difficult to identify in plain radiographs, which was the case in this patient. In a study, it was reported that the false-negative rate for plain films in detecting non-bony food bolus impaction without complications has been reported to be as high as 87% [11]. Another study reported that sensitivity of plain films in identifying sharp or pointed FBs is 42%-47% leading to the recommendation of multidetector CT scans which can improve detection reaching sensitivities of 97% [12]. Similarly, guidelines from the USA and Europe do not favour radiological imaging in case of non-bony food bolus impaction, but rather to proceed with endoscopy [10,11]. 

Prolonged impaction and delay in treatment of FB in an airway can lead to a wide variety of complications including obstruction and perforation of the oesophagus, mediastinitis, pneumothorax, pneumopericardium, tracheo-oesophageal, aorto-oesophageal and even oesophagobronchoaortic fistulas, aortic erosion, enterocolonic fistulas and colonic perforation [3]. Fortunately, our patient did not have any complications despite the prolonged history of denture impaction. We removed it successfully without any post-operative concerns. However, missed diagnoses on radiographs can have catastrophic outcomes. Similarly, a case was published in 2016, where a patient suffered prolonged asphyxiation and death following a prolonged impacted denture in oesophagus which was initially missed on plain radiographs [13]. These cases stress the importance of thorough and accurate clinical assessment including history taking, examination and correctly interpreting radiographic imaging, or opting for alternative imaging where appropriate. 

In the case of our patient, and considering his presentation and the above challenges discussed, diagnosis posed difficulty for the GP. Such a prolonged impaction without immediate life-threatening complications is very rare. We believe it is highly unlike a case similar to this has presented itself in the United Kingdom given the care pathways in place and availability of resources to help manage patients with FB impaction in the upper oesophagus and trachea.

## Conclusions

This case highlights the importance of CT neck in patients coming with a strong history of missing dentures and swallowing difficulty. Plain radiographs such as X-ray is not useful in case of denture impaction. Early diagnosis can help in immediate management and can prevent denture-associated complications in oesophagus. In addition, attempting a flexible upper GI endoscopy might be diagnostic and sometimes therapeutic. We also recommend using a Hopkins rod endoscopy along with a rigid oesophagoscope to improve the visualisation of the foreign body and aid in atraumatic removal. 

## References

[1] Sugawa C, Ono H, Taleb M, Lucas CE (2014). Endoscopic management of foreign bodies in the upper gastrointestinal tract: a review. World J Gastrointest Endosc.

[2] Nwaorgu OG, Onakoya PA, Sogebi OA, Kokong DD, Dosumu OO (2004). Esophageal impacted dentures. J Natl Med Assoc.

[3] Singh P, Singh A, Kant P, Zonunsanga B, Kuka AS (2013). An impacted denture in the oesophagus-an endoscopic or a surgical emergency-a case report. J Clin Diagn Res.

[4] Chen T, Wu HF, Shi Q (2013). Endoscopic management of impacted esophageal foreign bodies. Dis Esophagus.

[5] Litovitz TL, Klein-Schwartz W, White S (2001). 2000 annual report of the American Association of Poison Control Centers Toxic Exposure Surveillance System. Am J Emerg Med.

[6] Kerschner JE, Beste DJ, Conley SF (2001). Mediastinitis associated with foreign body erosion of the esophagus in children. Int J Pediatr Otorhinolaryngol.

[7] Abdullah BJ, Teong LK, Mahadevan J, Jalaludin A (1998). Dental prosthesis ingested and impacted in the esophagus and orolaryngopharynx. J Otolaryngol.

[8] Firth AL, Moor J, Goodyear PW, Strachan DR (2003). Dentures may be radiolucent. Emerg Med J.

[9] Karolyhazy K, Kivovics P, Fejerdy P, Aranyi Z (2005). Prosthodontic status and recommended care of patients with epilepsy. J Prosthet Dent.

[10] Ikenberry SO, Jue TL, Anderson MA (2011). Management of ingested foreign bodies and food impactions. Gastrointest Endosc.

[11] Birk M, Bauerfeind P, Deprez PH (2016). Removal of foreign bodies in the upper gastrointestinal tract in adults: European Society of Gastrointestinal Endoscopy (ESGE) clinical guideline. Endoscopy.

[12] Ma J, Kang DK, Bae JI (2013). Value of MDCT in diagnosis and management of esophageal sharp or pointed foreign bodies according to level of esophagus. AJR Am J Roentgenol.

[13] Funayama K, Fujihara J, Takeshita H (2016). An autopsy case of prolonged asphyxial death caused by the impacted denture in the esophagus. Leg Med (Tokyo).

